# In silico study of heterogeneous tumour-derived organoid response to CAR T-cell therapy

**DOI:** 10.1038/s41598-024-63125-5

**Published:** 2024-05-29

**Authors:** Luciana Melina Luque, Carlos Manuel Carlevaro, Enrique Rodriguez-Lomba, Enrique Lomba

**Affiliations:** 1 Centre for Regenerative Medicine, University of Edinburgh, Edinburgh, EH16 4UU UK; 2 Instituto de Física de Líquidos y Sistemas Biológicos, Consejo Nacional de Investigaciones Científicas y Técnicas, 1900 La Plata, Argentina; 3 Departamento de Ingeniería Mecánica, Universidad Tecnológica Nacional, Facultad Regional La Plata, 1900 La Plata, Argentina; 4 Hospital General Universitario Gregorio Marañón, 28007 Madrid, Spain; 5 Instituto de Química Física Blas Cabrera, Consejo Superior de Investigaciones Científicas, 28006 Madrid, Spain

**Keywords:** Computational models, Biological physics

## Abstract

Chimeric antigen receptor (CAR) T-cell therapy is a promising immunotherapy for treating cancers. This method consists in modifying the patients’ T-cells to directly target antigen-presenting cancer cells. One of the barriers to the development of this type of therapies, is target antigen heterogeneity. It is thought that intratumour heterogeneity is one of the leading determinants of therapeutic resistance and treatment failure. While understanding antigen heterogeneity is important for effective therapeutics, a good therapy strategy could enhance the therapy efficiency. In this work we introduce an agent-based model (ABM), built upon a previous ABM, to rationalise the outcomes of different CAR T-cells therapies strategies over heterogeneous tumour-derived organoids. We found that one dose of CAR T-cell therapy should be expected to reduce the tumour size as well as its growth rate, however it may not be enough to completely eliminate it. Moreover, the amount of free CAR T-cells (i.e. CAR T-cells that did not kill any cancer cell) increases as we increase the dosage, and so does the risk of side effects. We tested different strategies to enhance smaller dosages, such as enhancing the CAR T-cells long-term persistence and multiple dosing. For both approaches an appropriate dosimetry strategy is necessary to produce “effective yet safe” therapeutic results. Moreover, an interesting emergent phenomenon results from the simulations, namely the formation of a shield-like structure of cells with low antigen expression. This shield turns out to protect cells with high antigen expression. Finally we tested a multi-antigen recognition therapy to overcome antigen escape and heterogeneity. Our studies suggest that larger dosages can completely eliminate the organoid, however the multi-antigen recognition increases the risk of side effects. Therefore, an appropriate small dosages dosimetry strategy is necessary to improve the outcomes. Based on our results, it is clear that a proper therapeutic strategy could enhance the therapies outcomes. In that direction, our computational approach provides a framework to model treatment combinations in different scenarios and to explore the characteristics of successful and unsuccessful treatments.

## Introduction

Chimeric antigen receptor (CAR) T-cell therapy is a promising new immunotherapy that combines advances in cellular engineering and personalised medicine for patient-specific, targeted cancer treatment. This therapy involves collecting, purifying, and genetically modifying a patient’s own T-cells to express a CAR that specifically targets the patient’s tumour. These engineered cells are expanded ex vivo and then re-infused into the patient where the CAR T-cells target and kill antigen-expressing tumour cells^1–3^. So far, the FDA-approved CAR T-cell therapies and many studies expanding CAR designs exclusively target “liquid” cancers, against which they have shown great success in the clinic with response rates between 70–90%^4^. In contrast, response rates for solid cancers are significantly lower ranging from 4–16%^4^.

One of the barriers to the development of effective cellular therapies in solid tumours, specifically CAR T-cells, is target antigen intratumour heterogeneity^4,5^. Intratumour heterogeneity (also known as intralesion heterogeneity) refers to distinct tumour cell populations with different molecular and phenotypic profiles within the same tumour specimen^6,7^, and it is associated with poor prognosis and outcome^8–11^. It is thought that intratumour heterogeneity is one of the leading determinants of therapeutic resistance and treatment failure and one of the main reasons for poor overall survival in cancer patients with metastatic disease^7,12^. tumour heterogeneity has presented a considerable challenge to matching patients with the right treatment at the right time; therefore, it is a considerable handicap when it comes to accomplish the goals of precision medicine^13,14^.

One strategy to overcome antigen escape and heterogeneity is through the use of a multi-antigen recognition circuit involving complementary antigens^15,16^. One example of this is the syn-Notch receptor, which uses an engineered transmembrane receptor to induce expression of a tumour-specific CAR in response to recognition of an extracellular signal^16,17^. However, since tumour cells share antigens with other non-cancerous cells in the human body, to target the antigen that is specific to tumour cells and avoid normal human tissue has been a crucial challenge for the development of cellular therapies. While strategies such as those based on syn-Notch receptors are promising, great care has to be taken to find therapy strategies that will both be effective and minimally toxic to the patient.

Due to the interpatient variability and the genomic landscape of the disease, determining the outcome of therapies is not feasible in clinical settings. Instead, ex vivo models including classical monolayer cell culture^18^ and patient-derived xenografts^19^ have been used to study the effect of different treatments. In this regard, traditional cell culture models have had limited success because of their inability to recreate the complex interactions between the different cell types and the extracellular matrix (ECM). Although animal models have the advantage of providing the native three-dimensional (3D) microenvironment and multiorgan interactions, these models often fail to predict human responses to drugs as they do not accurately mimic human pathophysiology or the immune system.

Emerging advanced microengineering methods, including 3D cell culture platforms, have enabled scientists to develop new treatment strategies^20,21^. In this regard, as one of the powerful 3D tissue models, organoids offer a great opportunity to increase the scientific understanding of complex biology in a pathophysiological relevant condition^22,23^. Organoids have been used to establish living biobanks of cancer and normal tissues that preserve the genetic and functional heterogeneity among patients^24,25^. Moreover, tumour-reactive T-cells can be selectively expanded in co-culture with tumour organoids to study tumour-immuno interaction^26,27^.

However, exploring the multidimensional design space becomes prohibitively expensive and laborious, particularly when considering the time and resources required. Additionally, some design aspects and emergent properties are difficult to probe experimentally, such as cell-level behavioural states that impact treatment efficacy^28^. Employing in silico experiments has proven to be a resource-saving and valuable way to understand how underlying biological processes impact CAR treatment outcome and hypothesising new design features to improve efficacy^29^.

Within this broad context, a widely used modelling paradigm in the study of complex biological systems is the *agent-based model* (ABM)^30,31^. ABMs are implemented mainly to simulate the actions, behaviours and interactions of autonomous individual or collective entities, with the aim of exploring the impact of an agent or a type of behaviour in the system. An agent is the smallest unit in this model, and it can exhibit different types of behaviour, including interaction with other agents. Although these models simplify many aspects of reality, they have been shown to be extremely useful in a wide number of circumstances^32–34^. In cancer research, these models are emerging as valuable tools to study emergent behaviour in complex ecosystems^35^, and are used to study the mutational landscape of solid tumours^36,37^. Furthermore, they are increasingly used to optimise therapies, for example radiation therapy of solid tumours^38^. In immunotherapy, some models of immune-cell interactions have been proposed^29,39,40^. In particular, ABM that capture the temporal dynamics of effector T-cells and cancer cells during tumour progression in immune checkpoint inhibitors therapy^40,41^, are of great interest due to the similar characteristics that effector T-cells and CAR T-cells display. After primed in the lymph node, tumour neoantigen-specific naïve T-cells differentiate into effector T-cells, which can be recruited to tumour. These effector cells begin to exert their cytotoxic activity upon recognizing their antigen target in the tumour microenvironment. Some models also address the CAR T-cells interaction with immune cells, in order to investigate how clinically relevant design choices and inherent tumour features impact treatment outcomes^28,42^. Although these studies gave important insight into parts of the tumour-immune interaction, they do not fully investigate therapeutic strategies on heterogeneous tumours. By adjusting model parameters and simulation rules, the characteristics of successful and unsuccessful treatments can be explored to learn how therapy outcomes vary with a patient’s tumour characteristics^43–45^. Cancer immunotherapy could thus benefit from simultaneously employing molecular approaches (what medicinal chemistry can be employed to target specific molecular biology?) and multicellular systems-level approaches (what therapy protocol will lead to the best cancer control and induce remission?).

This work introduces a computational multiscale agent-based model to study immunosurveillance against heterogeneous tumour derived organoids, with a special focus on the spatial dynamics of stochastic tumour-immune contact interactions. It could predict the organoid response to different therapeutic strategies in order to discern whether a tumour is likely to respond to treatment or not. The model can be adjusted to reflect specific types of cancer to enable quantitative predictions of therapy-biomarker combinations and to be used as a platform for conducting virtual clinical trials.

## Results

### Less is better: increasing cellular dosage does not always increase efficacy

An important aspect of the CAR T-cell treatment is antigen selection. Since cancer share antigens with other non-cancerous cells in the human body, one of the major considerations when administrating CAR T-cell therapy is the potentially life-threatening side effects related to the number of (activated) CAR T-cells. Therefore, determination of the minimal number of CAR T-cells to be injected, such that the treatment is safe yet effective, is important. Such determination by trial and error can be lengthy, expensive, and inefficient. Moreover, CAR T-cell treatment involves complex dynamics of CAR T-cells—cancer interactions that make intuitive inference problematic, particularly when considering intratumour heterogeneity. Unlike experimental procedures, computer simulations allow for fast and precise evaluation of a large number of possible therapy regimens, including those that are difficult or costly to implement in vivo/in vitro.

We used our model to study different dosage strategies. Simulations take place in a grid of size $1000 \times 1000 \times 1000$ $\upmu \text {m}$. A spherical organoid of 3963 cells was seeded at the centre of the simulation box. To consider intratumour heterogeneity, each cell is assigned a mutant oncoprotein using a normal distribution that goes from 0 to 2 with a mean equal to 1 and a standard deviation of 0.25 (see “Methods” section below). Even though the oncoprotein expression is continuous, for practical reasons it was discretized in the plots. Cells are labelled to reflect their oncoprotein expression: Type 1 ($1.5 \le o < 2.0$), Type 2 ($1.0 \le o < 1.5$), Type 3 ($0.5 \le o < 1.0$), Type 4 ($0.0 \le o < 0.5$). Cell proliferation and immunogenicity scale proportional to *o*, and an oncoprotein expression lower than 0.5 is not enough to be recognised by T-cells.

**Figure 1:**
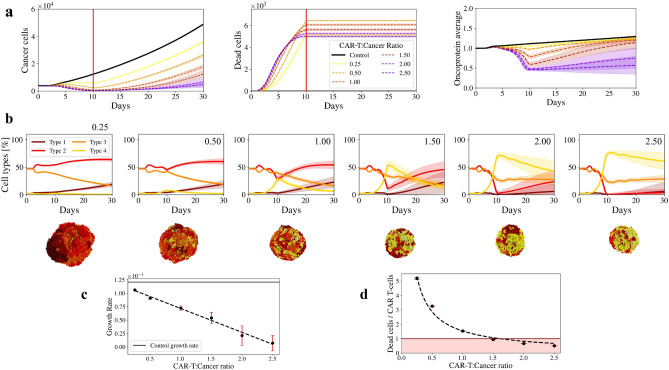
Heterogeneous tumour derived organoid response to different dosages of antigen specific CAR T-cell therapy. Organoid metrics over time, after applying different dosages of CAR T-cells. Dosages are represented by CAR T-cells to cancer cells ratios: 0.25, 0.50, 1.00, 1.50, 2.00, 2.50. The control case, i.e. untreated tumour, is represented by a full black line. Shaded regions represent the standard deviations of 20 simulations. Red line at day 10 represent CAR T-cell mean exhaustion time. (**a**) Left: Number of cancer cells. Centre: Number of dead cells, Right: Average of the oncoprotein expressed in the organoid. (**b**) Top plots show the percentage of the cell types that constitutes the organoid after applying the different dosages of immunotherapy. The oncoprotein expression in the cells is continuous, but for practical reasons it was discretized in the plots. Cells are labelled to reflect their oncoprotein expression: Type 1 ($1.5 \le o < 2.0$), Type 2 ($1.0 \le o < 1.5$), Type 3 ($0.5 \le o < 1.0$), Type 4 ($0.0 \le o < 0.5$). Type 1 represent the most mutated cells, which means higher proliferation rate and higher probability of dying from an immune attack. Type 4, on the other hand, are those cells that cannot be killed, because the mutation burden is not enough to be recognised by the CAR T-cells. Their proliferation rate is very low. Below the plots there are schematic representations of the organoids at the day 30. (**c**) Organoid growth rate after therapy, for every different dosages. Control case is represented by a solid black line. (**d**) Dead cancer cell per CAR T-cell ratio for the different dosages. Red area highlights the cases for which the killing ratio is less than 1, meaning that there are CAR T-cells that never kill a cancer cell.

As shown in Fig. 1a left, an organoid without treatment (black solid line) grows fast due to the fact that the cells with higher oncoprotein expression, i.e. the most proliferative cells, dominate its dynamics. It can be seen in Fig. 1a right, which shows the organoid mean oncoprotein expression value. By the end of the simulation this value went from 1.00 to to 1.31, meaning that, despite the initial state of the organoid in which oncoprotein was normally distributed, it will evolve into a rapidly growing organoid.

To examine the efficacy of the CAR T doses, we performed focused simulations in which different dosages of CAR T-cells at various T-cell-to-cancer ratios were applied at day 1. The ratios range from 0.25 to 2.50. CAR T-cells perform a biased random migration towards an immunostimulatory gradient to find cancer cells. While adhered to a target cell, the immune cell agent attempts to induce apoptosis with a probability that scales linearly with immunogenicity. If successful, the tumour cell undergoes apoptosis, while the immune agent detaches and resumes its chemotactic search for additional tumour cell targets. If the immune cell does not kill the tumour cell, it remains attached while making further attempts to induce apoptosis until either succeeding or reaching a maximum attachment lifetime, after which it detaches without inducing apoptosis. In our model, T-cells have a stochastic lifespan with the exhaustion time mean at day 10 (please refer to “Methods” section below for more details).

Figure 1a left shows that the number of cancer cells decreased after the treatment was introduced and before the CAR T-cells exhaustion time (solid red line). We can observe that the organoid shrinks as we increase the T-cell to cancer ratio, however, ratios larger than 1.00 resulted in no significant difference as all the curves fall within each other standard deviations. After the exhaustion time the organoid expands rapidly for smaller ratios. To further understand that behaviour, we can look at the organoid oncoprotein average (Fig. 1a right). Before exhaustion time, the oncoprotein average dramatically decreased for ratios larger than 1.00. Ratios 2.00 and 2.50 almost reached an average of 0.50, which is the value for which the mutations present in cancer cells are not enough for CAR T-cells to recognise them, although there is no significant difference between them. If we look deeper into the cell types distribution within the organoids for the different experiments (Fig. 1b), we can see that small ratios are not enough to eliminate Type 1 and 2 cells (the more proliferative cells) and therefore, they take over the organoid. We can observe that in a quantitative manner in the plots at the top in which we analyse the percentage distribution of each cell type within the organoid during the entire simulation time. We can also observe the cell type distribution at the last day of the simulation in a qualitatively way in the screenshots at the bottom. If we compare ratios 0.25 and 2.50, Type 4 cells are more abundant in ratio 2.50, explaining the low oncoprotein average, the slowing down in the organoid growth after the exhaustion time and also the small number of cancer cell deaths. If we look at the accumulated value of dead cells in the centre plot of Fig. 1a we can see that by the exhaustion time, ratios 0.25 and 2.50 killed the same amount of cells. However, ratio 2.50 rapidly killed most of Type 1, 2 and 3 cells, and due to the fact that CAR T-cells cannot kill Type 4 cells, it reached a plateau before the exhaustion day. On the other hand, ratio 0.25, killed as many cells as ratio 2.25 by the exhaustion day, but because it did it slower, Type 1 cells had time to proliferate and took over the organoid. We can quantify this behaviour by plotting the organoid growth rate for each dosage (Fig. 1c). We can see that larger ratios drastically reduce the growth rate compared with the non treated organoid (black solid line).

After this analysis the logic conclusion would be that it is better to apply doses of larger ratios. However, since one of the major considerations when administrating CAR T-cell therapy is the potentially side effects related to the number of free CAR T-cells, we also looked at the ratio between dead cells and CAR T-cells (Fig. 1d). We found out that for ratios larger than 1.00, there were a large number of CAR T-cells that never killed a cancer cell. Our computational model suggests a possible explanation to this phenomenon, which relies on the organoid geometry and heterogeneity. Since CAR T-cells reach the organoids by its exposed surface, once the first CAR T-cells reach the organoid, they generate a layer that prevent the next CAR T-cells to reach the cancer cells. Whenever the first group of CAR T-cells finishes its attempt to kill the cancer cells, most of the cells that are left for the second group of CAR T-cells to kill are Type 4 cells, i.e., cells that cannot be killed by CAR T-cells. That means that most of the CAR T-cells that are following the first group, will not kill a cancer cell. The amount of free CAR T-cells increases as we increase the dosage. This result agrees with other studies that indicate that increasing dose does not result in a maximal rate of killing on a per T-cell basis^46,47^.

Based on the dead cells/CAR T-cells ratio and on the fact that ratios larger than 1.00 have no significant difference on cancer cells and on the average oncoprotein value before the exhaustion day, our model suggests that a ratio of 1.00 is the ideal “effective yet safe” dosage to treat a heterogeneous organoid. This agrees with murine experiments in which the estimated ratio between CAR-T and tumour cells was 1:1^47^.

Finally, increasing the CAR T-cell number does not necessarily increase the killing ratio, however, higher ratios reduce the mutational load of the tumour, making it less prolific by the end of the simulation. One would like to reach those results without the problem of the free CAR T-cells. Current studies suggest that CAR T-cell “effective yet safe” approach might be improved by enhancing smaller doses. One possible strategy to improve the activity of CAR T-cells is to increase CAR T-cell persistence to prevent exhaustion^48,49^. Another strategy might be to provide multiple doses of highly active CAR T-cells to replace those that have become hypofunctional^5^. We will discuss both approaches in the following sections.

### Long-term persistence might not be needed

One of the major goals in CAR T-cell therapy research is to generate CAR T-cells with the longest possible persistence in order to minimise the extent of CAR T-cells infusion^48,49^. To further analyse the effect of long-term persistent CAR T-cells, we apply the same dosages as before, but this time the CAR T-cells last the entire simulation time.

**Figure 2:**
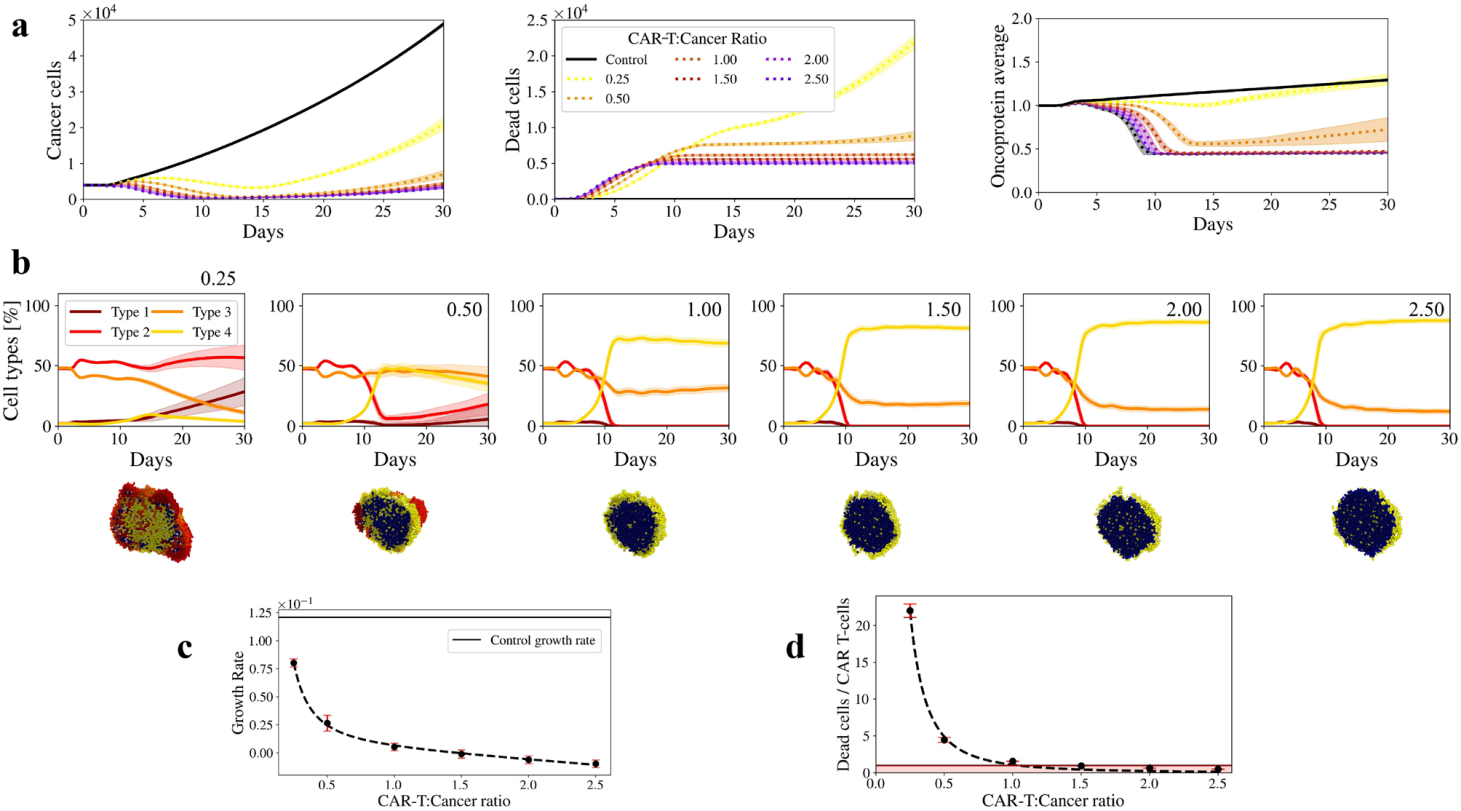
Heterogeneous tumour response to long-term persistent CAR T-cells. We apply the same dosages as before, but this time the CAR T-cells lasted the entire simulation time. The control case, i.e. untreated tumour, is represented by a full black line. Shaded regions represent the standard deviations of 20 simulations. (**a**) Left: Number of cancer cells. Centre: Number of dead cells. Right: Average of the oncoprotein expressed in the organoid. (**b**) Top plots show the percentage of the cell types that constitutes the organoid after applying the different dosages of immunotherapy. Below the plots, the schematic representations show the organoid resection at the day 30. Blue cells represent CAR T-cells. (**c**) Organoid growth rate after therapy, for every different dosages. Control case is represented by a solid black line. (**d**) Dead cancer cell per CAR T-cell ratio for the different dosages. Red area highlights the cases for which the killing ratio is less than 1, meaning that there are CAR T-cells that never kill a cancer cell.

In Fig. 2a left we can see that enhanced CAR T-cells have a greater impact than non-enhanced ones. Ratios greater than 1.00 still significantly reduce the tumour growth as well as its average mutation rate (Fig. 2a right), but this time we also see a drastic reduction in ratios smaller than 1.00. Ratios 0.25 and 0.50 reduce the tumour growth compared to the previous case, but cannot completely eliminate the most mutated cells. Since the killing rate of CAR T-cells is lower than the proliferation rate of cancer cells, the organoids keep on growing. Interestingly, while CAR T-cells try to kill cells in the centre of the organoid, cancer cells proliferate on the surface. Particularly, for the ratio 0.50, an emergent behaviour due to the intratumour heterogeneity is that a layer of Type 4 cells grows on the surface of the organoid and the most mutated cells that could not be killed by the CAR T-cells, proliferate on the surface of such layer, which prevents CAR T-cells from killing them (Fig. 2b).

Another interesting aspect is that for dosages of ratio greater than 1.00, the tumour growth rate reaches a plateau (Fig. 2c), indicating that tumour growth is the same regardless of the number of CAR T-cells. However, in Fig. 2d we see that the efficiency of each CAR T-cell is notably reduced for ratios greater than 1.00. This tells us that, as in the previous section, most of the CAR T-cells remain free. To make matters worse, in this case they have an extended life, which increases the risk of side effects.

Finally, our model suggests that enhancing the smaller doses, as we hypothesised in the previous section, reduces the growth rate and tumour mutation compared to the previous case. However, smaller ratios result in CAR T-cells spatially distributed in an unfavourable manner, which reduces their function and, therefore, the efficiency of the therapy. On the other hand, larger ratios result in free CAR T-cells, which increases the risk of side effects. That gives us the hint that enhancing the persistence of the CAR T-cells will not necessarily improve the therapy outcomes, but it can be associated with increasing levels of T-cell hypofunction due to unfavourable spatial distribution and/or low dead cells to CAR T-cell ratios. This is in agreement with previous studies that indicates that clustering of CAR T-cells results in their exhaustion and therefore, a reduction in the therapy efficiency^46,50,51^.

### Multiple dosing to prevent hypofunction

**Figure 3:**
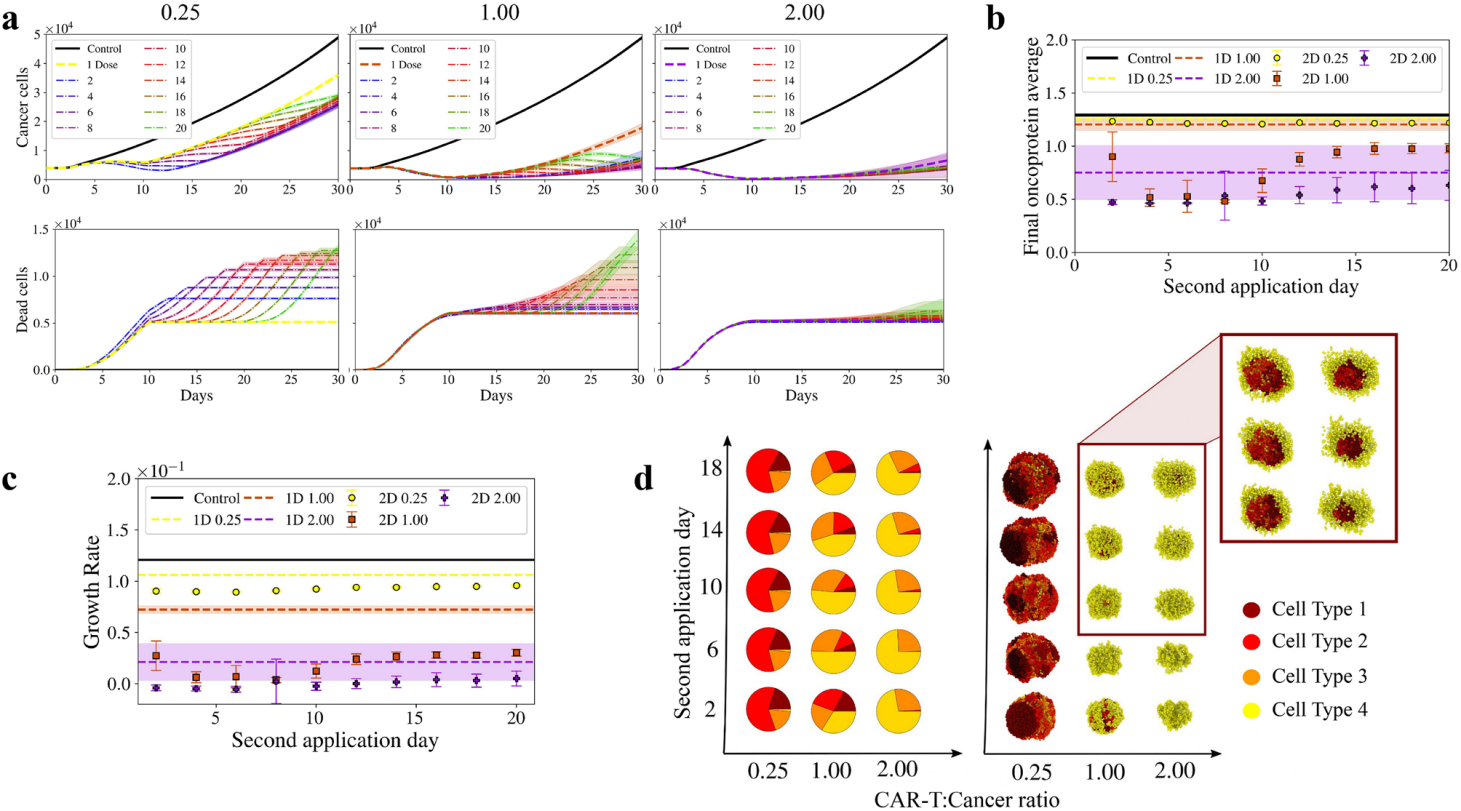
Heterogeneous organoid response to multiple dosing strategies. We apply second doses of CAR T-cell therapy at different days from the first dose: 2, 4, 6, 8, 10, 12, 14, 16, 18, 20. Dosages correspond to ratios 0.25, 1.00, 2.00, and first and the second dose contain the same amount of T-cells. Shaded regions represent the standard deviations of 20 simulations. (**a**) Upper panels show the number of cancer cells for dosages of different ratios, while lower panels show the number of dead cells. (**b**) Average of the oncoprotein expressed in the organoid in terms of the day of the second dose application. Black solid line and dashed lines represent the average oncoprotein value expressed by the organoid on the $30{\text {th}}$ day of the simulation, in control case and organoids treated with only one dose of CAR T-cells respectively. Shaded regions represent the standard deviation. Scatter points represent the average oncoprotein value expressed by the organoid on the $30^{th}$ day of the simulation, after two doses of immunotherapy. Error bars represent the standard deviation. (**c**) Organoid growth rate computed by the $30{\text {th}}$ day of the simulation, in terms of the day of the second dose application. Black solid line and dashed lines represent control case and organoids treated with one dose of immunotherapy, respectively. Scatter points represent the organoid growth rate after two doses of immunotherapy. Shaded regions and error bars represent the standard deviation. (**d**) Left: Pie charts showing the percentage of the cancer cell types that constitutes the organoid after applying two doses of immunotherapy. Type 1 represent the most mutated cells (higher proliferation rate and higher probability of dying from an immune attack), whereas Type 4 cells, on the other hand, are those cells that cannot be killed, because the mutation burden is not enough to be recognised by the CAR T-cells. Y-axis represent the day of the second dose application, and X-axis contain the different dosages. Right: Schematic representation of the organoids after two doses of immunotherapy. The organoids framed were resectioned to show the spatial organisation of the different cell types.

Another alternative to enhance small dosage of CAR T-cell therapy, and also avoid unfavourable spatial distribution, can be to provide multiple doses of highly active CAR T-cells to replace those that have become hypofunctional. We apply second doses of CAR T-cell therapy at different days from the first dose. The first and the second dose contain the same amount of T-cells. As we can see in Fig. 3a left, for doses of ratio 0.25, at the end of the simulation (day 30), a second dose (dashed dotted lines) has a similar impact on cancer cells compared to the impact that a first dose (dashed line) has on the non-treated organoid. If we look at the efficiency of the second dose before the exhaustion days (10 days after the application), doses applied before the $8{\text {th}}$ day, reduced the organoid size, while doses applied after that, delayed the organoid growth but weren’t capable of reducing its size. If we look at the organoid behaviour after the exhaustion time, the application day does not make much difference in terms of cancer cells, i.e. at day 30 all of the curves collapse. However, in terms of dead cells, the more we wait to apply the second dose, the more cells the CAR T-cells kill. This outcome can be related to a larger exposed area.

If we look at ratio 1.00 (Fig. 3a centre), the first dose already reduced the organoid size and killed most of the proliferative cells, causing early applications of the second dose to be less effective. We can see that in the Dead cells plot, in which doses applied before the $8{\text {th}}$ day barely killed a cancer cell. The two applications of ratio 1.00 dosage before the exhaustion day have a similar effect in terms of cancer cells. But at the end of the simulation, the second dose has a significant smaller impact than the first dose. Similar to case 0.25, late applications produce more dead cells. Finally, applying two doses of ratio 2.00 do not make any difference compared to one dose.

If we look at the tumour oncoprotein average at day 30 (Fig. 3b), when applying just one dose (dashed lines), only the dose of ratio 2.00 shows a significant reduction on the oncoprotein load of the tumour, compared to the control case (solid black line). When applying two doses (scatter points), the dose of ratio 0, 25 stays close to the control and the one dose cases, and remains almost constant across the application days. A second dose of ratio 1.00 shows a significant reduction compared to the application of only one dose, particularly when applied between days 4 and 8. Surprisingly, when applied at day 8, its effect is even better than the effect of a second dose of ratio 2.00. We can see a similar behaviour when looking at the growth rate (Fig. 3c). The largest reduction between first and second dose happens when applying two doses of ratio 1.00, with the second one applied on the $8{\text {th}}$ day.

The fact that the second dose, particularly in high doses, does not have the same impact as the first one on the size of the tumour but it has a high impact on the oncoprotein average and the growth rate, results from the fact that the first dose eliminated most of type 1 cells, which are the most likely to be killed by T-cells. This can be seen qualitatively in Fig. 3d, in which for a low dose such as 0.25, organoids are larger and present a high percentage of Type 1 cells, independently of the second dose application day. For higher doses, the therapy reduces the organoid size and the percentage of Type 4 cells (cancer cells that cannot be killed by T-cells) increased from one dose to another.

**Figure 4:**
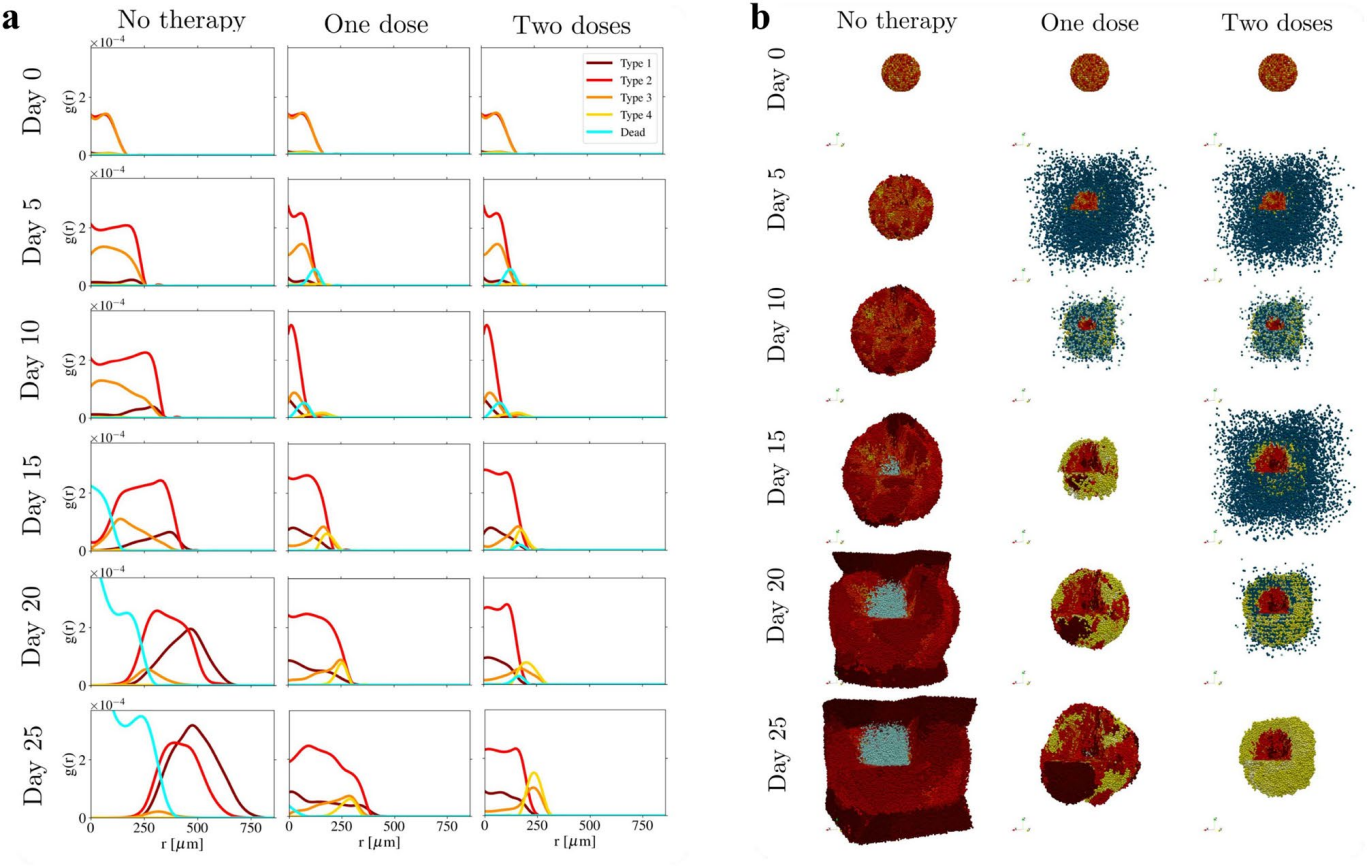
Shield-like structure formation. (**a**) Radial distributions, *g*(*r*), of different types of cells, in terms of the distance from the centre of mass of each organoid to its surface. Left column shows the control case, while the centre column and the right column shows an organoid treated with one dose and two doses of CAR T-cell therapy respectively. Dosage corresponds to ratio 2.00. Type 1 cells are plotted in dark red, Type 2 in red, Type 3 in orange and Type 4 in yellow. Cyan curves represent dead cells, whether they have died for a T-cell attack or for lack of oxygen. (**b**) 3D plot of the progression of a non treated organoid (left), and an organoid treated with one (centre) and two (right) doses of CAR T-cell therapy, on specific days. T-cells are shown in dark blue, dead cells are shown in light blue. At day 25, after two doses of CAR T-cell therapy one can observe that a shield-like structure of cells with low oncoprotein expression has formed over cells with high oncoprotein expression. This leads to a reduction of therapy efficiency. Animations of the heterogeneous organoid response to one and two doses of antigen specific CAR T-cell therapy can be seen in the Additional Information S1 Video.

Additionally, the use of a second dose of CAR T-cells gave rise to an interesting emergent phenomena. Type 4 cells form a shield-like structure that prevents Type 1 and Type 2 cells to be reached by T-cells. To have a quantitative approximation of this behaviour, Fig. 4 shows the radial distribution *g*(*r*) of the different cell types inside the organoid. The distance *r* ranges from the centre of mass of each organoid, to its surface and is divided in spherical shells of width, $\Delta r$, of about 5 cells radii. Day 25 shows clearly how Type 1 and Type 2 cells (i.e. the more proliferative cells) take over the tumour dynamics, whereas after two doses of immunotherapy those cells substantially decrease in number and a shield of Type 3 and Type 4 cells forms around them. This is the main reason why second doses of ratios larger than 1.00 loose their efficiency.

This agrees with several discussions about the importance of the spatial distribution in heterogeneous tissues and how this can hamper the efficiency of different immunotherapies^52^. If one could only look at the reduction of the organoid size or at the growth rate, the logic decision would be to apply another dose of immunotherapy. However, if we look at the spatial distribution of the cells we can predict that a third dose will not be efficient unless a complementary therapy that eliminates the shield of Type 4 cells is previously applied. Moreover, a third dose of immunotherapy will result in free CAR T-cells that enhance the chances of side effects and increases costs renders the treatment inefficient. Moreover, before applying the CAR T-cell therapy the patient must receive chemotherapy in order to deplete his/her immune system. This carries the risk of infection and bleeding and is thus not suitable all patients. These potential complications should be avoided unless the therapy’s effectiveness fully justifies it.

### Heterogeneous tumour response to multi-antigen recognition CAR T-cell therapy

These strategies are promising as regards the goal of reducing the CAR T-cell dosage and enhance its efficiency. However, due to the antigen heterogeneity, CAR T-cells are not able to fully eliminate the organoid, which tends to relapse with low-antigen presenting cells. We also observed that in some cases, low-antigen cells form a shield like structure that protects antigen rich cells from CAR T-cells, reducing the therapy efficiency. Thus, there is a general need for multiple antigen recognition strategies that can overcome the challenges of heterogeneity to increase the therapeutic benefit of CAR T-cells^5^. With that in mind, we tested, a multi-antigen recognition type of therapy, such as syn-Notch receptor. In this approximation, T-cells can target every cancer cell, regardless of its oncoprotein expression value. However they can also attack healthy cells, therefore one should be careful when planning the dosimetry strategy.

**Figure 5:**
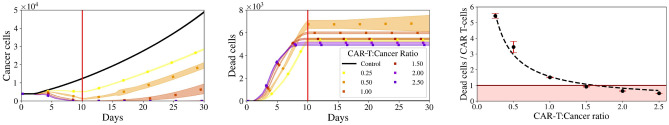
Heterogeneous tumour response to multi-antigen recognition. Organoid metrics over time, after applying different dosages of a multi-antigen recognition type of therapy such as syn-Notch receptor. As in the previous cases, dosages are represented by different ratios: 0.25, 0.50, 1.00, 1.50, 2.00, 2.50. The control case, i.e. untreated tumour, is represented by a full black line. Shaded regions represent the standard deviations of 20 simulations. Red line at day 10 represent CAR T-cell mean exhaustion time. Left: Number of cancer cells. Centre: Number of dead cells. Right: Dead cancer cell per CAR T-cell ratio for the different dosages. Red area highlights the cases for which the killing ratio is less than 1, meaning that there are CAR T-cells that never kill a cancer cell.

Figure 5 shows the main results of our strategy compared to a non treated organoid (black line). Simulation suggests that doses of ratio larger than 1.50, will successfully eliminate the organoid. Since one of the goals of this type of therapies is their capacity to target cancer cell not only in the primary tumour but in the whole body, this result is very promising if one considers that one might be dealing with a smaller, early stage metastasis that was not diagnosed. Doses of ratios smaller than 1.50 show a reduction in the organoid growth, but it is not enough to completely eliminate it. On the other hand, if we look at the Dead cells/CAR T-cells ratio, we see that the efficiency of each CAR T-cell is notably reduced for ratios greater than 1.50. This tells us that, as in the previous sections, most of the CAR T-cells remain free, an undesired result which is worsened by the multi-antigen recognition feature. All these details increase the risk of side effects. Our simulations suggests that the ideal “effective yet safe” dosages are those of ratio smaller than 1.50. However, even though these ratios show a reduction in tumour growth, they do not suffice to completely eliminate it. Once again, an appropriate dosimetry strategy is necessary to produce effective therapeutic results.

## Discussion

One of the barriers to the development of effective cellular therapies, specifically for CAR T-cells, is target antigen heterogeneity. It is thought that intratumour heterogeneity is one of the leading determinants of therapeutic resistance and treatment failure. While understanding antigen heterogeneity is important for effective therapeutics, a good therapy strategy could enhance the therapy efficiency.

In order to develop effective CAR T-cell strategies, physiological preclinical models are required that recapitulate the individual tumour phenotype as well as the complex three-dimensional tissue environment. Organoids allows long-term ex vivo expansion of different cell types in a 3D extracellular matrix. The technology has been used to establish living biobanks of cancer and normal tissues that preserve the genetic and functional heterogeneity among patients. Even though they lack two key aspects on tumour development, such as different cell types populations that are present in the tumour microenvironment and blood vessels, they proved to be a good platform for Cancer—CAR T-cells crosstalk. Unfortunately, patient derived organoids are difficult to grow due to cell type heterogeneity and also present challenges associated with their cost and ethics.

Within this broad context, the aim of this work is to introduce an agent-based model in order to rationalise the potential outcomes of CAR T-cell therapies over patient derived heterogeneous tumour organoids, using a computational approach. The importance of computational models lies at its ability to predict non-intuitive results. Here, we show that using an ABM model, we are able to analyse the results of different treatments characterised by different schedules and dosages, without wrecking the organoid with therapies that are not likely to produce any significant outcome.

We started our study by analysing different dosages in order to determine the optimal “effective yet safe” CAR T-cell to Cancer ratio. Our model suggests that increasing the CAR T-cell number does not necessarily increase the killing ratio. Since CAR T-cells reach the organoids by its exposed surface, this phenomenon is related to the organoid geometry and heterogeneity. In that sense, it is important to mention that even though we present spherical organoids in this work, the geometry is one of the parameters that can be adjusted in our model, not only as an input parameter but also during the execution time. Higher ratios reduce the mutational load of the tumour making it less prolific, but by the end of the simulation, the amount of free CAR T-cells increases as we increase the dosage, and so does the risk of side effects. We found out that a ratio of 1.00 is the ideal dosage to treat a heterogeneous organoid. It shows a considerably reduction in tumour size as well as in its growth rate, but most important, free CAR T-cells do not occur. This agrees with murine experiments in which the estimated ratio between CAR-T and tumour cells was 1:1^47^.

We then tested two possible strategies to improve the activity of CAR T-cells with particular interest in smaller ratios. On one hand we increased the CAR T-cells persistence to prevent exhaustion. We found that due to the antigen heterogeneity, enhancing small ratios persistence result in CAR T-cells spatially distributed in an unfavourable manner, which reduces their function and, therefore, the efficiency of the therapy. On the other hand, larger ratios result in free CAR T-cells, which increases the risk of side effects. That gives us the hint that enhancing the persistence of the CAR T-cells will not necessarily improve the therapy outcomes, but it can be associated with increasing levels of T-cell hypofunction. This outcome is the opposite to what one would expect due to the general consensus that the successes of CAR T-cells in patients with certain haematological cancers are closely linked with CAR T-cell persistence. However, the importance of persistence has not yet been established in clinical trials testing CAR T-cells in patients with solid tumours, for a number of reasons. Firstly, given the lack of clinical success in patients with solid tumours, along with uniformly short persistence, establishing correlations between CAR T-cell persistence and efficacy, as has been done in trials involving tumour infiltrated lymphocytes and with CAR T-cells in leukaemias and lymphomas, has not been feasible. Secondly, the relationship between persistence in blood and persistence in solid tumours has not yet been fully validated. Thirdly, and perhaps most importantly, data from solid tumour models indicate that CAR T-cell persistence is associated with increasing levels of T-cell hypofunction, as we observe in our model. Please refer to the recent review by Albelda^5^ for a insightful discussion on this topic.

The other strategy was to provide multiple doses of highly active CAR T-cells to replace those that have become hypofunctional. A second dose was applied at different days, for different dosage ratios. The tumour size was reduced as well as the tumour growth rate. Also, our model suggests that a second dose of ratio 1.00 shows a significant reduction compared to the application of only one dose, particularly when applied between days 4 and 8, i.e. not very late but also not very soon. Surprisingly, when applied at day 8, its effect is even better than the effect of a second dose of ratio 2.00. Nevertheless, in terms of dead cells, second doses turned out to be less effective than the first dose. Computational outcomes suggests that this lack of efficiency might be due to the fact that the first dose eliminated most of the high-oncoprotein expressing cells. Since immunogenicity scales proportional to the oncoprotein expression, *o*, T-cells either do not recognise low-oncoprotein expressing cancer cell, or spend more time trying to kill them (sometimes without success).

One emergent phenomenon that came out of the simulations, and might be another reason for therapy inefficiency in solid tumours, is the formation of a shield-like structure of cells with low oncoprotein expression and reduced proliferation rate, that protected cells with high oncoprotein expression. It has been discussed in several reviews that CAR T-cell infiltration into the tumour is a major roadblock for its success in solid tumours^42,53^, and that various cell types have been found to prevent CAR T-cell infiltration as well as a dense extra cellular matrix^53,54^. For instance, another in silico model has found a similar behaviour when applying a binary heterogeneity antigen approach^55^. In a recent study, Baldominos et al.^54^, observed a similar effect in triple negative breast cancer, in which tumour cells that resist T-cell attack are quiescent, i.e. the key genes involved in proliferation and DNA replication, such as Mki67 and Pcna, were all downregulated. Similar to our Type 4 cancer cells. Quiescent cancer cells (QCCs) form micro-niches with reduced immune infiltration. The authors adapted single-cell RNA-sequencing with precise spatial resolution to profile infiltrating cells inside and outside the QCC niche. This transcriptomic analysis revealed hypoxia-induced programs and identified more exhausted T-cells, tumor-protective fibroblasts, and dysfunctional dendritic cells inside clusters of QCCs. This uncovered differential phenotypes in infiltrating cells based on their intra-tumour location. Thus, QCCs constitute immunotherapy-resistant reservoirs by orchestrating a local hypoxic immune-suppressive milieu that blocks T-cell function. Although the complex biological mechanisms that lead to the formation of the niches found by Baldominos, are simplified in our model, in both cases it is clear that the development of strategies to overcome immune suppression around QCCs/Type 4 cancer cells and eradicate these cells will be key to improving responses to immunotherapy and preventing recurrence after treatment.

In order to overcome antigen escape and heterogeneity, another approach of therapy, based in the syn-Notch receptor, has been studied. In this context T-cells can target every cancer cell, regardless of its oncoprotein expression value. It has been found that doses of ratio larger than 1.50, will successfully eliminate the organoid. Since one of the milestones of this type of therapies is their capacity to target cancer cell not only in the primary tumour but in the whole body, this result is very promising if one consider this large ratio as an early stage metastasis—CAR T-cell ratio. In other words, If we apply a dose of a small ratio compared to the primary tumour, but the patient developed an early stage metastasis, i.e. a smaller tumour, the normal ratio will become a large ratio compared to the metastasis and will successfully eliminate it. Since cancers share antigens with other non-cancerous cells in the human body, great care has to be taken to find therapy strategies that will both be effective and minimally toxic to the patient. Our simulations suggests that the ideal “effective yet safe” dosage are those of ratio smaller than 1.50. However, even though these ratios show a reduction in tumour growth, they are not enough to completely eliminate it, so an appropriate dosimetry strategy is necessary to produce effective therapeutic results.

There are several limitations of this model which point towards new directions for further development. One of the main constraints for its widespread use is the computational cost of the model. Even though thread parallelisation in relevant sections of the algorithm is currently implemented, a full graphic processing units oriented re-writing of the most time consuming parts of the code is desirable. This will enhance the model’s capacity to reach time-space scales that are unattainable so far. From a more practical standpoint, at this stage the model has not been calibrated to any patient-specific cancer. This an obvious handicap for its direct application in the clinical practice. Clearly, a future line of work will have to focus on to tuning of model parameters to reproduce the behaviour of a patient derived organoid. In this way, it will serve as a tool for hypotheses testing in the planning of alternative therapeutic protocols.

Finally, a few words concerning our model’s implementation. We have been particularly careful to construct a software platform in a modular and extensible fashion. The aforementioned modules can be replaced with ones with more fine-grained versions as discussed, so that more specific details can be incorporated (as properties) and new processes as well (as methods) with different degrees of detail. Even though our model is not a 1 : 1 in silico copy of the organoid and, therefore, it can not accurately describe in full detail the complex biological processes, it could serve as a tool to test different hypotheses, as well as for testing and analysing possible outcomes using multiple plausible parameter combinations. We are confident that once the goal of implementing patient specific factors is reached and the model undergoes a rigorous calibration and validation, it could be used as a platform for in silico conducting virtual clinical trials.

## Methods

The model presented herein builds upon previous work by Luque et al. on tissue growth kinetics^56^. The following subsections will briefly recall details of the mentioned model. Please, refer to the previous reference for further details. Subsequently in subsections Intratumour heterogeneity and Immunosurviellance, we will comment on the new features related to intratumoural heterogeneity and immunosurviellance modules implemented in this work.

### Model setup

Our model is implemented resorting to an object oriented programming model, and to that aim C++11 language have been used. Simulation CPU time depends on model parameters such as domain (lattice) size, cell number and simulation length (in time); a typical simulation run takes approximately 6 h on a single core of an Intel i7-10510U CPU. Model visualisation is performed with Ovito^57^, Paraview^58^ and Matplotlib^59^.

### Diffusion solver

Cell behaviour is mostly dependent on the values and gradients of diffusing substrates in the tumour microenvironment. Diffusion process is modelled as a vector of reaction-diffusion partial differential equations for a vector of chemical substrates. It is discretized over a Cartesian mesh for computational convenience, in such a way that each voxel (volumetric pixel) stores a vector of chemical substrates. Each substrate diffuses and decays, and can be secreted or uptaken by individual cells at their specific positions.

To model the effect of blood vessels, which are not used in this work but can be easily added to the simulations, or to apply Dirichlet boundary conditions, the so-called Dirichlet nodes are also implemented. In that implementation, substrate values at any voxel within the simulation domain can be overwritten to turn the voxel into a continuous source of substrates.

### Cell agents

**Figure 6:**
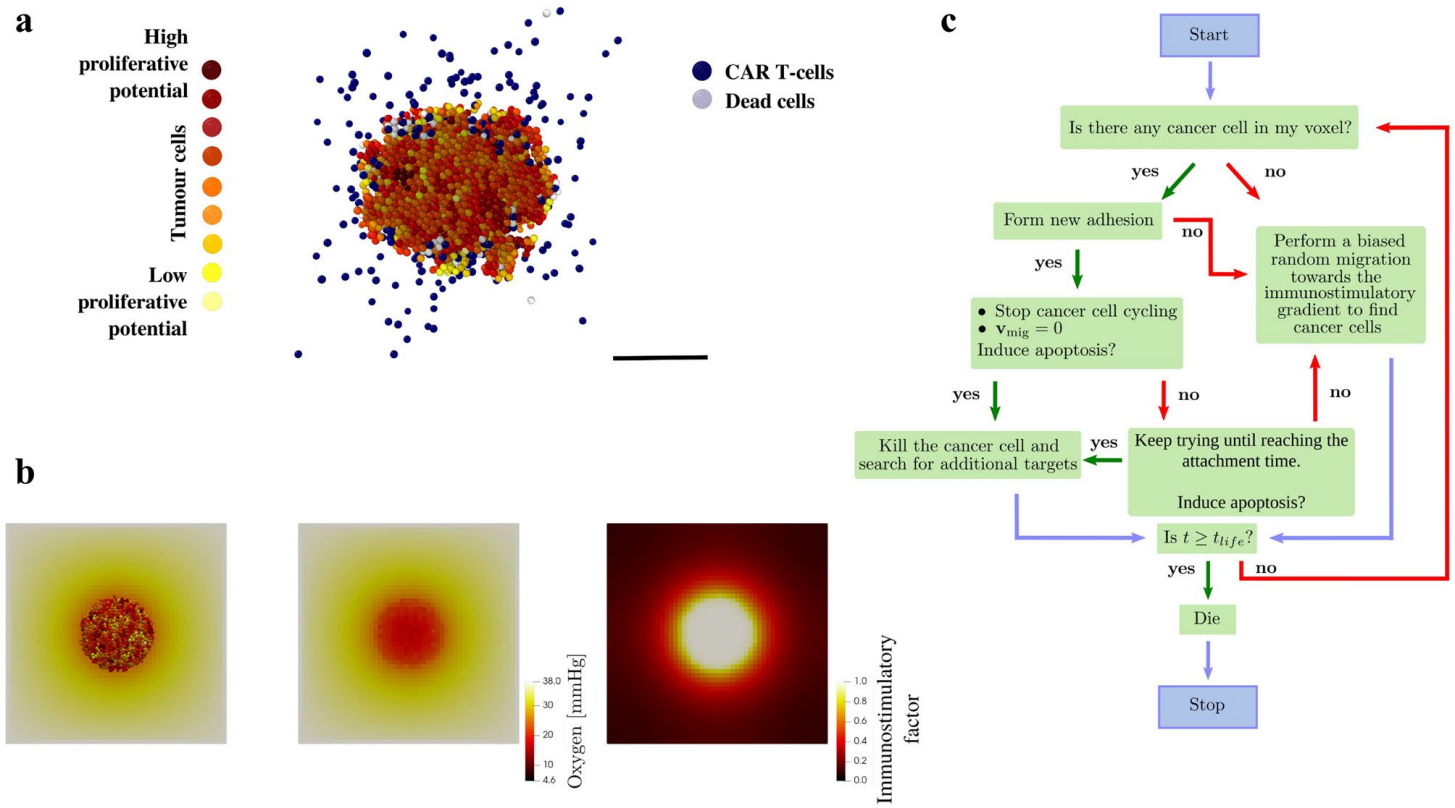
Immunosurviellance process. Immune cells perform a biased random migration towards an immunostimulatory gradient to find cancer cells. (**a**) Schematic representation of an organoid being attacked by immune cells. Scale bar represent $200\, \upmu m$. (**b**) An heterogeneous organoid consuming oxygen (mmHg) from the microenvironment, and secreting an immunostimulatory factor (in arbitrary units). (**c**) Immunosurviellance flow diagram. $t_{life}$ represents the CAR T-cell exhaustion time.

In the context of cancer immunology, the agents represent cancer and immune cells (Fig. 6a). Their motion is governed by the balance of adhesive, repulsive, motile, and drag-like forces. It is important to note that repulsive forces are really an elastic resistance to deformation.

One of the main features that makes our model different from others in the literature is that cells are off-lattice. Consequently, they are not confined to a particular lattice or spatial arrangement, they move through all space positions, and therefore underlying possible artifacts associated with the chosen lattice structure and spacing are removed.

Each cell has an independent cell cycle which is modelled as a directed graph, and can also progress through apoptotic and necrotic death processes. Any of the cell cycle (and death processes) time scales can be adjusted at the beginning of the simulation to match different types of growth and they can also be adjusted at any time on an individual cell in order to reflect the influence of its microenvironment.

As the cell progresses through its current cycle, it varies its volume (and sub volumes, such as nuclear volume, solid volume, fluid volume, etc.). These volumes are modelled with a system of ordinary differential equations that allow cells to grow or shrink towards a target volume.

As it was mentioned earlier, each cell can secrete to or uptake from its chemical microenvironment, or sample the value or gradient of any or all substrates. This is very important since most of the cellular processes depend on the substrates that diffuse in the microenvironment. In every simulation step, each cell checks the substrate concentration in its voxel and base its behaviour upon them. Figure 6b shows an organoid consuming oxygen from the microenvironment, and secreting an immunostimulatory factor. This is one of the most important data structures of the cell because it links the cell with its microenvironment. Its inner workings are modelled by a vector of partial differential equations which in practice implies the addition of a cellular secretion/uptake term to the diffusion equation described in section: “Diffusion solver”.

### Intratumour heterogeneity

Even though eukaryotic cells replicate their DNA with astounding fidelity, the mechanism is not entirely error free. Every time a cell divides, a few mutational errors in the form of nucleotide substitutions and small deletions are introduced even in the absence of internal and external mutagens^60,61^. Owing to the constant turnover of tumour cells and the large size of tumour cell populations, some of these stochastic mutational hits unavoidably affect genes with known cancer relevance, leading to the activation of oncogenes and/or inactivation of tumour suppressors, such as the p53 gene. The TP53 gene provides instructions for making a protein called tumour protein p53 (or p53). This protein acts as a tumour suppressor, which means that it regulates cell division by keeping cells from growing and dividing (proliferating) too fast or in an uncontrolled way.

Among the many factors that drive tumour heterogeneity, genomics instability is most prominent in all malignancies. Many of the biological hallmarks associated with cancer development, such as limitless replicative potential, increase the mutational rate and genomics instability of malignant cells, which in turn give rise to other malignant traits^62–64^. This cascading effect often results in heterogeneity in the tumour as different cells acquire unique mutations that give rise to genetically distinct subpopulations^65–68^.

To study intratumour heterogeneity, each cancer cell is provided with a random expression of a mutant “oncoprotein”, *o*, using a normal distribution (a similar computational approach could be made to model intratumour heterogeneity based on the inactivation of the tumour suppressor p53 gene). This oncoprotein drives proliferation, i.e. the greater the expression of *o*, the more likely the cell cycles and divides. In the absence of other selective pressures, the cells with the greatest *o* expression clonally expand and dominate the dynamics of the tumour. Under the simplifying assumption that a highly-expressed mutant protein would be reflected as a more immunogenic peptide signature on major histocompatibility complexes (MHCs)^69^, each cell’s immunogenicity is modelled as proportional to *o*.

### Immunosurviellance

To model immunosurveillance T-cell agents are introduced. One of the main difference between T-cells and cancer cells present in our model, is that the former are self-propelled. In other words, in addition to the forces due to the interaction with other cells and the basement membrane, immune cells move in response to chemical stimuli. As it was mentioned before, cancer cells secrete an immunostimulatory factor which diffuses through the microenvironment. Immune system cells perform a biased random migration towards this immunostimulatory gradient to find cancer cells. The migration performed along the direction **d**, which is updated according the immunostuimulatory factor gradient, is governed by the bias *b*, which can take values $0 \le b \le 1$ where 0 means Brownian motion and 1 represents deterministic motion along **d**. Immune system cells change their migration velocity stochastically between *t* and $t + \Delta t_{\text {mech}}$ with probability $\Delta t_{\text {mech}}/t_{\text {per}}$, where $t_{\text {per}}$ is the CAR T-cell’s mean persistence time. To change the velocity a random direction, $\textbf{d}_r$, is chosen by $\textbf{d}_r = \left[ \sin { \left( \phi \right) } \cos { \left( \theta \right) }, \sin { \left( \phi \right) } \sin { \left( \theta \right) , \cos { \left( \phi \right) } } \right]$, where $\theta$ is a random angle between $\left[ 0, \pi \right]$ and $\phi$ is a random angle between [$0, 2\pi$]. The migration velocity $\textbf{v}_{mig}$ is then updated according to

1 $$\begin{aligned} \textbf{v}_{\text {mig}} = v_{\text {mot}}\frac{(1-b)\textbf{d}_{r} - b\textbf{d}}{|| (1-b)\textbf{d}_{r} - b\textbf{d} ||} \end{aligned}$$

where $v_{\text {mot}}$ is the migration speed. Notice that if the migration bias *b* is 1, the CAR T-cell will perform a deterministic motion over the immunostimulatory factor gradient direction $\textbf{d}$, while on the other hand, if $b=0$, it will perform a Brownian motion over the random direction $\textbf{d}_{r}$. If the immune cell is attached to a cancer cell, its velocity is set to zero. Finally, when updating the immune cell’s velocity, its migration velocity $\textbf{v}_{\text {mig}}$ is added to the current velocity computed by the interaction with other cells.

T-cells continuously test for contact with cancer cells. In fact, if they detect contact, in any time interval, they have a probability of forming an adhesion regulated by $r_{\text {adh}} \Delta t$, where $r_{\text {adh}}$ is the rate of forming new adhesions. Once they form an adhesion they switch off their motility and cancer cells stop their cycling activity. While adhered to a target cell, the immune cell agent attempts to induce apoptosis (e.g., by the FAS receptor pathway^70^) with a probability that scales linearly with immunogenicity. In the multi-antigen recognition case, the immunogenicity is not taking into account, and the probability of inducing apoptosis follows a uniform distribution. If successful, the tumour cell undergoes apoptosis, while the immune agent detaches and resumes its chemotactic search for additional tumour cell targets. If the immune cell does not kill the tumour cell, it remains attached while making further attempts to induce apoptosis until either succeeding or reaching a maximum attachment lifetime, after which it detaches without inducing apoptosis. Scanty information is available regarding the functional and/or immunophenotypic characteristics of the CAR T-cells after infusion^71^. CAR T-cell exhaustion is thought to be due to persistent antigen stimulation, as well as an immunosuppressive tumour microenvironment^72^. Previous studies observed that CAR T-cells are only detected for about a month after infusion, with a peak typically seen at 5–14 days^5,71^. To capture such dynamics, exhaustion time is stochastic in our model, with a mean of exhaustion time, $t_{\text {life}} = 10$ days, meaning that some CAR T-cells can get exhausted before or after the $10{\text {th}}$ day, but most of them will do it at the $10{\text {th}}$ day. In our model, when a CAR T-cell gets exhausted it is removed from the simulation, similar to dead cells. A schematic representation of the inner working of CAR T-cells is depicted in Fig. 6c.

### Reference tables

In this section we provide the table that enumerates the model parameters (Table 1). The goal of this work is to develop a computational platform to study how clinically relevant design choices and inherent tumour features impact treatment outcomes. Even though the cancer cell cycle times correspond to those of the hepatocellular carcinoma in order to follow up with our previous study, we do not limit our model to represent any specific cancer type at this stage, thus model calibration and parametrization is qualitative at this stage. Values of parameters were taken from the literature, from experimental studies or adopted from previous models if they apply to a range of cancers. For those parameters for which we are unable to find an experimental value, or those that differ across cancer types, we estimate a range based on best biological knowledge. In addition to that, most of the parameters used in this work were used were used in a previous study on tissue growth kinetics. In that occasion we performed a sensitivity analysis in which we varied $10\%$ the input variables that feed our model in order to find out which parameters were most likely to make an impact on the tumour growth. We invite the reader to delve into the sensitivity analysis and the parameter choice of our model in our previous work^56^.

**Table 1:** Input parameters for the model and references for each parameter. Some of the parameters had been estimated in previous studies^56^.

Parameter	Description	Value	Units	References
Time parameters				
$T_{Tot}$	Total simulation time	43200	min	–
$t_{save}$	Saving time	1400	min	–
$\Delta t_{cycle}$	Cell cycle time step	6	min	^73^
$\Delta t_{mech}$	Cell mechanics time step	0.1	min	^73^
$\Delta t_{diff}$	Diffusion time step	0.01	min	^73^
$t_v$	Update voxel lists of particles time	$20\Delta t_{mech}$	min	–
Simulation domain and diffusion parameters				
$\Omega _{x}, \Omega _{y}, \Omega _{z}$	Domain size	$1000 \times 1000 \times 1000$	$\upmu$m	–
$\Delta x, \Delta y, \Delta z$	Microenvironment voxel size	$20 \times 20 \times 20$	$\upmu$m	–
$n_{x}, n_{y}, n_{z}$	Mechanics discretization voxel size	$30 \times 30 \times 30$	$\upmu \text {m}$	–
$D_{O_{2}}$	Oxygen diffusion coefficient	$10^{5}$	$\upmu \text {m}^{2}/\text {min}$	^74^
$\lambda _{O_{2}}$	Oxygen decay rate	0.1	$\text {min}^{-1}$	^75^
$D_{IF}$	Immunostimulatory factor diffusion coefficient	$10^{3}$	$\upmu \text {m}^{2}/\text {min}$	^73^
$\lambda _{IF}$	Immunostimulatory factor decay rate	0.016	$\text {min}^{-1}$	^73^
Cancer cell parameters				
$n_{\text {div}}$	Cell divisions allowed	$\infty$	dimensionless	–
*p*	Division polarization	0	dimensionless	–
$T_{G1}$	Duration of $G_{1}$ phase	20.4	h	^76^
$T_{S}$	Duration of *S* phase	13.6	h	^76^
$T_{G2}$	Duration of $G_{2}$ phase	3.0	h	^76^
$T_{M}$	Duration of *M* phase	1.6	h	^76^
$R_{A}$	Maximum cell adhesion distance	$1.25R_{cell}$	$\upmu \text {m}$	^77^
$C_{ccr}$	Cell–cell “repulsive” force constant	$10.0\nu$	$\upmu \text {m}/\text {min}$	^78^
$IF_S$	Immunostimulatory factor release rate	10	$\text {min}^{-1}$	–
$O_U$	Oxygen uptake rate	10	$\text {min}^{-1}$	^56^
CAR T-cell parameters				
$n_{\text {div}}$	Cell divisions allowed	0	dimensionless	–
$r_{K}$	Kill rate	0.06	$1/\text {min}$	^79^
$t_{a}$	Adhesion time	60	min	^43^
$r_{a}$	Adhesion rate	0.2	$1/\text {min}$	^43^
$R_{LA}$	Maximum adhesion distance	18	$\upmu \text {m}$	^73^
$C_{ccr}$	Cell–cell “repulsive” force constant	$5.0\nu$	$\upmu \text {m}/\text {min}$	^73^
$O_U$	Oxygen uptake rate	1	$\text {min}^{-1}$	–
$t_{life}$	Lifespan mean (±SD)	10 ($\pm 5$)	$\text {days}$	^71^
$T_{per}$	Migration persistence time	10	min	^79^
$v_{\text {mot}}$	Mean migration speed	2	$\upmu \text {m}/\text {min}$	^80^
*b*	Migration bias	0.5	dimensionless	^81^

### Supplementary Information

Supplementary Information.

## Data Availability

The datasets used and/or analysed during the current study available from the corresponding author on reasonable request.

## Code availability

The code used for running experiments is available at https://github.com/lmluque/abm.
