# Supplementary material for: In silico study of heterogeneous tumour-derived organoid response to CAR T-cell therapy
Source: Sci Rep. 2024 May 29;14:12307. doi: 10.1038/s41598-024-63125-5 (PMC11137006; doi:10.1038/s41598-024-63125-5)
Supplement: Supplementary file 1 — Supplementary Information. [file 41598_2024_63125_MOESM1_ESM.docx]

**Supplementary information.** S1 Video. 3D simulation of the heterogeneous organoid response to one and two doses of antigen specific CAR T-cell therapy . Video available at https://youtu.be/nyK98yZdQSs.
